# Outcomes of 243 dogs with traumatic fractures treated with the I‐Loc interlocking nail

**DOI:** 10.1111/vsu.14320

**Published:** 2025-07-28

**Authors:** Karen Lisette Perry, Rachel Wesslen

**Affiliations:** ^1^ Pat Carrigan Professor of Feline Health Michigan State University East Lansing Michigan USA; ^2^ Michigan State University East Lansing Michigan USA

## Abstract

**Objective:**

To describe clinical application and outcomes following stabilization of traumatic long‐bone fractures in dogs using an I‐Loc angle stable interlocking nail (AS‐ILN).

**Study design:**

Retrospective study.

**Sample population:**

A total of 243 client‐owned dogs.

**Methods:**

The medical records of dogs with long‐bone fractures stabilized with an I‐Loc AS‐ILN were reviewed. Data collected included signalment, affected bone, fracture pattern and whether fractures were open/closed. Surgical details included nail parameters, customization, use of ancillary implants and number of missed bolts. Complications were classified as major or minor depending upon requirement for revision surgery. Time to clinical union was documented.

**Results:**

A total of 243 fractures affected the femur (138/243), tibia (71/243), humerus (27/243) or radius/ulna (7/243). Most were diaphyseal (183/243) and comminuted (151/243). The 8, 7, 6, 5, 4 and 3 mm nails were used in dogs weighing an average of 41, 30, 26, 21, 13 and 5 kg, respectively. Ancillary implant use was uncommon (50/243). Nails were shortened in 40/243 cases and prebent in 55/243. The postoperative missed bolt rate was 8/852. Radiographic follow‐up was achieved in 189 cases, with a median duration of 57.5 days. Mean time to clinical union was 8.3 weeks. Major and minor postoperative complications were encountered in 13/243 and 22/243 of cases, respectively.

**Conclusion:**

Use of the I‐Loc system was associated with positive results across a wide range of presentations including epi−/metaphyseal fractures.

**Clinical significance:**

The low complication rates associated with the I‐Loc system indicate potential benefits to patients compared to plate‐rod systems, non‐angle‐stable nails or other AS‐ILNs.

**Abbreviations**
AS‐ILN: Angle stable interlocking nail
LCP: Locking compression plate
ORIF: Open reduction and internal fixation
MINO: Minimally invasive nail osteosynthesis
SSI: Surgical site infection

## 1 INTRODUCTION

Due to inherent mechanical and biological advantages, interlocking nails have become an attractive alternative to bone plating or plate‐rod constructs for stabilization of long‐bone fractures.^1^, ^2^, ^3^, ^4^ Compared with matching bone plates, interlocking nails have a relatively larger and more uniform area moment of inertia which allows them to effectively resist bending.^5^, ^6^ Furthermore, their intramedullary position near the neutral axis of the bone, protects them from deleterious bending moments. Interlocking nails also provide axial and rotational stability via locking bolts or bone screws.

Interlocking nails are often used in people because they can be placed in a closed fashion hereby protecting the extraosseous blood supply and enhancing biological fracture fixation.^7^ Similarly, in dogs, interlocking nails can be placed using minimally invasive techniques, a technique known as minimally invasive nail osteosynthesis or MINO.^8^

As a result of mechanical deficiencies which resulted in persistent rotational and bending instability,^3^, ^4^, ^6^, ^9^ traditional interlocking nail designs were associated with moderately high complication rates including prolonged healing times, excessive callus formation and a need for supplementary implants.^1^, ^10^, ^11^, ^12^ This instability was attributed to the absence of a rigid mechanical interlock between the nail and the locking device.^3^ To circumvent such limitations, angle‐stable interlocking nails (AS‐ILN) have been developed which eliminate both torsional and bending slack and provide superior clinical results.^4^, ^13^, ^14^, ^15^, ^16^, ^17^, ^18^

There are three AS‐ILNs currently commercially available for use in small animals; the I‐Loc (BioMedtrix, New Jersey),^4^, ^13^, ^14^, ^15^, ^16^, ^17^ the Targon (B Braun Vet Care, Germany),^19^, ^20^ and the Surg'X (Surg'X, France).^18^, ^21^ The I‐Loc is available in diameters from 3.0 to 8.0 mm, the Surg'X from 3.5 to 8.0 mm and the Targon in 2.5 and 3.0 mm.

The success rate of interlocking nailing using non‐angle‐stable nails varies from 83% to 96%, with healing times ranging from 13 to 17 weeks.^1^, ^22^, ^23^, ^24^ Complications are reported in up to 17% of cases treated.^23^ Although most are related to the nail being used when poorly indicated (such as for treatment of metaphyseal fractures), some may be attributed to limitations of nail design including implant fracture and the development of delayed and non‐union.^8^

There is a paucity of information regarding clinical outcomes following use of AS‐ILNs in dogs. A prospective case series detailing the results of 30 long‐bone fractures in cats stabilized using the I‐Loc AS‐ILN demonstrated minor complications in two out of 30 cases (6.6%) with no major complications.^17^ The mean time to clinical union was 7.2 weeks. A recent update on this report detailed an additional 12 cases with a maintained absence of major complications.^25^ Early clinical experience with the Targon nail detailed results in 49 cats and eight small‐breed dogs with a major complication rate of 9/57 (15.8%) and a minor complication rate of 3/57 (5.3%).^20^ Clinical experience with the Surg'X nail in 38 dogs and 52 cats was documented in 2024.^18^ The major complication rate was 7/61 (11.5%) with no minor complications reported.

While satisfactory clinical results have been reported with the Targon nail,^20^ this system is only available in two sizes appropriate for cats or small breed dogs. Additionally, as the bolts with this system must be placed at the junction between the metaphysis and the diaphysis, indications for use are limited to diaphyseal fractures only. While early evaluation showed promising results, subsequent studies demonstrated that locking mechanism slippage between the intramedullary rod and the set screws can lead to a recurrence of torsional instability.^26^

The Surg'X nail addresses these concerns related to the Targon nail; however, there may be some concerns about the use of titanium for this implant; titanium has a yield strength which is very close to the ultimate strength which can render implants more likely to fracture than deform.^27^ Also, titanium is very notch sensitive which could lead to fatigue failure.^27^ If the nail is scratched or damaged during insertion, which unfortunately with off‐axis bolt placement remains a possibility, then this could create a localized focal point which could lead to crack initiation and propagation from cyclic loading. A further consideration is the hollow shaft of this implant; this will decrease the area moment of inertia and increase compliance. In a recent report, three of the six major complications reported using the Surg'X nail involved implant fracture at the third proximal screw hole within 15 days postoperatively.^18^

Given the limitations and persistent concerns regarding alternative AS‐ILN systems, it is possible that the I‐Loc system may provide advantages. The I‐Loc is available in six different diameters, in lengths from 62 to 235 mm and is manufactured from 316L stainless‐steel. However, clinical results following use of the I‐Loc in dogs remain limited to an abstract detailing 100 traumatic fractures stabilized using this system.^16^ The major complication rate in this report was 3/100 (3%) and the minor complication rate 7/100 (7%). Complete follow‐up was achieved in 59 cases with all achieving clinical union and returning to normal function, but limited additional data is available.

Increased detail regarding clinical application and outcomes following use of AS‐ILNs, particularly the I‐Loc nail, will allow more definitive recommendations to be made regarding their use and determine where each system may be clinically advantageous. As such, the objective of this study was to document clinical application and outcomes of consecutively treated traumatic long‐bone fractures in dogs using the I‐Loc AS‐ILN system and to consider the implications of these for treatment of future cases.

## 2 MATERIALS AND METHODS

As a retrospective study of clinical cases which was purely observational, no ethical approval for this study was required. The database of a single referral hospital was searched for dogs that had undergone stabilization of traumatic fractures of the femur, humerus, tibia or radius/ulna using an I‐Loc AS‐ILN. The 100 cases detailed within the previous abstract^16^ were included but were re‐evaluated specifically for this study. Anesthesia and analgesia protocols varied but all were developed by board‐certified or board‐eligible anesthesiologists. Revision surgeries and pathologic fractures were excluded. All other fractures were included in the initial stage of data collection regarding clinical application; however, only those with follow‐up to clinical union were included in analysis of radiographic outcome. Retrieved data included signalment and presence of any orthopedic comorbidities. Comorbidities encountered at least twice were detailed specifically, while injuries only encountered once were grouped together as other. Fracture chronicity and fracture laterality were recorded.

Pre‐and postoperative orthogonal radiographs were reviewed by a board‐certified surgeon (KLP) and fractures classified in terms of the bone affected, fracture location (proximal articular, proximal metaphyseal, diaphyseal, distal metaphyseal or distal articular) and fracture pattern (transverse, short oblique, long oblique, spiral, segmental, comminuted but reconstructable or comminuted and non‐reconstructable). Fractures were additionally classified as either open or closed.

The surgical approach was classified as MINO or open reduction and internal fixation (ORIF). Nail diameter and length were documented. Nail diameter in relation to dog bodyweight was recorded. Any nail customization was noted; specifically, whether the nail tip was shortened to allow maximal bolt purchase in the distal cancellous shelf and whether the nail was bent to accommodate retro‐ or procurvatum. The plane in which the bolts were placed was recorded as an indication of nail orientation, in addition to any variations from the standard 2:2 bolt configuration. The use of ancillary implants was noted.

Any missed nail cannulations were recorded, including which specific bolt locations were affected. Missed bolts were classified as those missed but detected and retrieved intraoperatively, and those missed and only detected on postoperative radiographs.

Presence of postoperative complications was recorded and specific details of the complication noted. Complications were defined as any undesirable outcome associated with the surgical procedure and were classified as major (surgical intervention performed) or minor (managed without surgical intervention).

For all cases where radiographic follow‐up was available, these were reviewed by a board‐certified surgeon and time to clinical union was recorded.^28^ Additionally, in cases where this was stated specifically in the medical records, time to lameness resolution was noted.

Depending upon outcome measure, data are reported as either absolute number and percentage or mean ± SD and/or range.

## 3 RESULTS

### 3.1 Signalment

A total of 243 traumatic long‐bone fractures were evaluated. The most common breeds were mixed breed (*n* = 74), Labrador Retriever (*n* = 47), American Pit Bull Terrier (*n* = 19), German Shepherd Dog (*n* = 15), Golden Retriever (*n* = 8), Boxer (*n* = 6) and Siberian Husky (*n* = 6). A total of 40 other breeds were represented with between one‐to‐five dogs in each category. The mean age was 42.7 months ± 41.2 months (range: 3–168 months) and mean bodyweight was 29.8 ± 11.8 kg (range: 3.26–76 kg).

Orthopedic comorbidities were present in 66/243 (27.2%) cases; 40 cases suffered one comorbidity, 23 cases suffered two, and three cases suffered three leading to a total of 95 comorbidities. These are detailed in Table 1. The mean chronicity of the fracture at the time of surgery was 2.9 days ± 3.0 days (range: 0–32 days).

**TABLE 1:** Specific details of orthopedic comorbidities encountered in 243 traumatic long bone fractures stabilized using angle‐stable interlocking nails.

Orthopedic comorbidity	Number encountered
Pelvic fractures	25
Contralateral femoral fracture	8
Contralateral coxofemoral luxation	7
Bilateral stifle osteoarthritis ± instability in cranial drawer	7
Bilateral hip osteoarthritis	5
Ipsilateral radius/ulna fracture	4
Ipsilateral tibial fracture	4
Ipsilateral scapular fracture	4
Rib fractures	4
Ipsilateral coxofemoral luxation	3
Ipsilateral elbow luxation	3
Ipsilateral femoral head/neck fracture	3
Contralateral tibial fracture	2
Contralateral metatarsal fractures	2
Contralateral femoral head/neck fracture	2
Multiple maxillofacial fractures	2
Other	10

### 3.2 Bone distribution and fracture pattern

A total of 110 fractures out of 243 (45.3%) affected the left side and 133 (54.7%) the right. There were 138/243 (56.8%) femoral fractures, 71 (29.2%) tibial fractures, 27 (11.1%) humeral fractures and seven (2.9%) radius/ulna fractures stabilized using an AS‐ILN within the ulna. Fractures were classified as diaphyseal in 183 (75.3%) cases, metaphyseal in 53 (21.8%) and articular in seven (2.9%). Fracture pattern classification is shown in Table 2 but overall, 82/243 (33.7%) fractures were classified as comminuted but theoretically reconstructable, 69 (28.4%) as comminuted and non‐reconstructable, two (0.8%) as segmental and the remaining 90 (37.0%) as simple. A total of 23 fractures (9.5%) were classified as open.

**TABLE 2:** Fracture pattern classification of 243 traumatic long bone fractures stabilized using angle‐stable interlocking nails.

Fracture pattern classification	Number of fractures	Percentage of fractures
Comminuted but reconstructable	82	33.7
Comminuted, not reconstructable	69	28.4
Long oblique	28	11.5
Spiral	26	10.7
Transverse	22	9.1
Short oblique	14	5.8
Segmental	2	0.8

### 3.3 Surgical procedure

ORIF was employed in 152/243 (62.6%) cases while 91 (37.4%) cases were stabilized via MINO. When considering nail diameter, 3 mm nails were used in five cases, 4 mm nails in 16, 5 mm nails in 20, 6 mm nails in 67, 7 mm nails in 73 and 8 mm nails in 62 cases. The mean bodyweight of dogs receiving each nail diameter is shown in Table 3. One dog was removed from these calculations due to atypical nail application creating an outlier; this was a 3 mm nail used to stabilize an ulnar fracture in a 44.4 kg dog.

**TABLE 3:** Mean, SD and range of bodyweights of dogs receiving each angle‐stable interlocking nail diameter.

Nail diameter	Number of dogs	Mean and SD bodyweight (kg)	Range of bodyweight (kg)
8 mm I‐Loc	62	41.1 ± 11.6 kg	22.0–76.0 kg
7 mm I‐Loc	73	30.8 ± 6.6 kg	11.2–47.0 kg
6 mm I‐Loc	67	26.1 ± 7.2 kg	15.6–45.2 kg
5 mm I‐Loc	20	20.7 ± 6.8 kg	12.5–35.1 kg
4 mm I‐Loc	16	13.2 ± 6.2 kg	5.84– 25.45 kg
3 mm I‐Loc	4	5.4 ± 2.0 kg	3.26–8.0 kg

**FIGURE 1:**
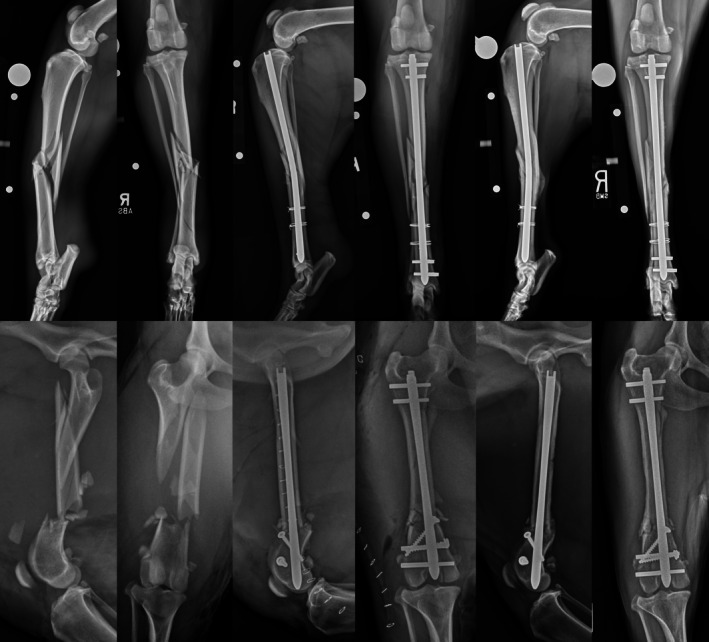
Use of ancillary implants. Top row – Preoperative, immediate and 6‐week postoperative radiographs of a 6‐year‐old mixed breed dog with a comminuted mid‐diaphyseal tibial fracture with fissures extending distally. The fracture was stabilized using a 8 × 235 mm I‐Loc AS‐ILN in 2:2 configuration with two double loop cerclage wires. Bottom row – Preoperative, immediate and 10‐week postoperative radiographs of a 6‐year‐old mixed breed dog with a comminuted mid‐to‐distal diaphyseal femoral fracture with fissures extending to the articular surface. The fracture was stabilized using 7 × 160 mm I‐Loc AS‐ILN in 2:2 configuration with two 2.7 mm lag screws to control distal fissures.

No ancillary implants were used in 193/243 (79.4%) cases. When used, the most common was double‐loop cerclage wires, placed in 41 (16.9%) cases. Position or lag screws were used in three (1.2%) cases to control fissures, both screws and double‐loop cerclage were used in three (1.2%) cases (Figure 1). A locking compression plate (LCP) was used in two (0.8%) cases and a tension‐band wire in one (0.4%) case. The cases where LCPs were used included a 76 kg Mastiff with a tibia/fibula fracture and a 44 kg Doberman Pinscher with a radius/ulna fracture where the ulnar AS‐ILN was used in conjunction with a radial LCP. Ancillary implants were more commonly used in epi−/metaphyseal fractures (33/60; 55%) than in diaphyseal fractures (17/183; 9.3%).

**FIGURE 2:**
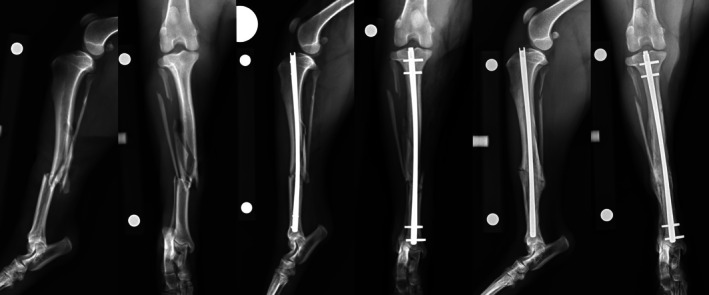
Nail modifications. Preoperative, immediate and 6‐week postoperative radiographs of a 4‐year‐old Beagle with a Gustilo‐Anderson type II open, chronic, comminuted fracture of the mid‐tibial diaphysis. The fracture was stabilized using a 4 × 126 mm I‐Loc AS‐ILN in 2:2 configuration with modification of the tip of the nail to allow deep seating.

**FIGURE 3:**
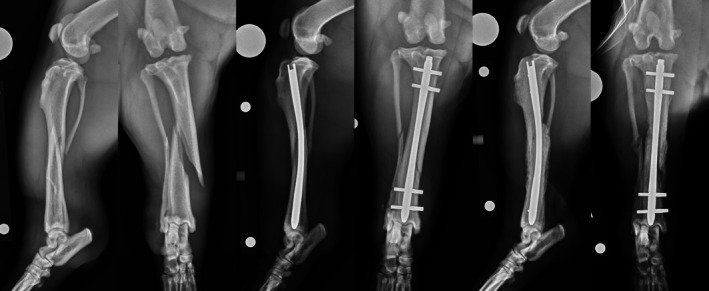
Nail bending. Preoperative, immediate and 6‐week postoperative radiographs of a 1‐year‐old Pitbull with a Gustilo‐Anderson type I open long oblique fracture of the mid tibial diaphysis. The fracture was stabilized using a 6 × 122 mm I‐Loc AS‐ILN in 2:2 configuration bent to an angle of 168° to follow tibial recurvatum.

In 40/243 (16.5%) cases, the nail was shortened using a lathe as previously described by Marturello et al.^17^ to facilitate deep seating within the distal metaphysis. This was performed in 12 of 71 tibiae (16.9%) (Figure 2), 14 of 138 femora (10.1%), 13 of 27 humeri (48.1%) and one of seven ulnae (14.3%). Nail prebending to follow natural retro‐ or procurvatum was performed in a total of 55/243 (22.6%) cases (Figure 3). The most common indication was for tibial fracture stabilization; 46/71 (64.8%) tibial nails were prebent, 6/138 (4.3%) femoral nails, no humeral nails and 3/7 (42.9%) ulnar nails. Nail orientation was altered in 9/243 (3.7%) cases to avoid fissures and facilitate bolt engagement. This was performed in 4/71 (5.6%) tibiae, 4/7 (57.1%) ulnae and 1/27 (3.7%) humerus; in all cases the nail was placed to facilitate placement of the bolts in a craniocaudal direction.

**FIGURE 4:**
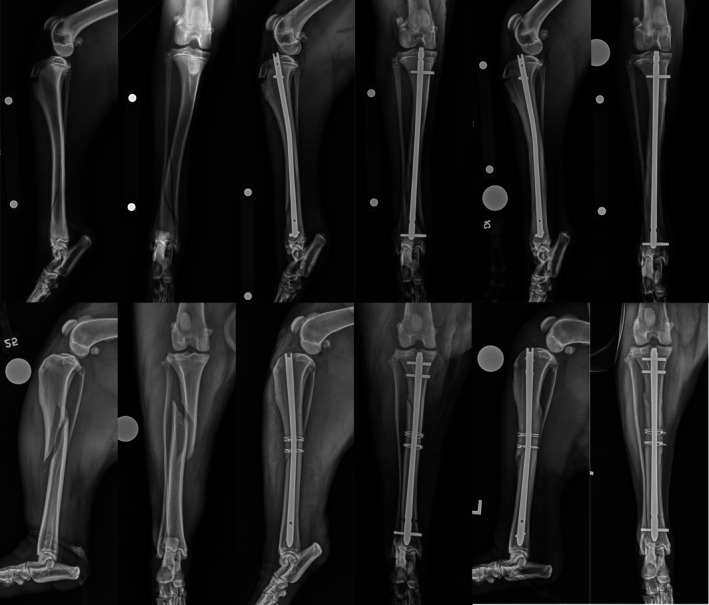
Bolt configurations. Top row – Preoperative, immediate and 3‐week postoperative radiographs of a 5‐month‐old German Shorthaired Pointer with a spiral fracture of the distal tibial diaphysis. The fracture was stabilized using a 6 × 185 I‐Loc AS‐ILN in 1:1 configuration via minimally invasive nail osteosynthesis (MINO). Bottom row – Preoperative, immediate and 6‐week postoperative radiographs of a 2‐year‐old Labrador Retriever with a long oblique fracture of the mid‐to‐proximal tibial diaphysis. The fracture was stabilized using a 7 × 185 mm I‐Loc AS‐ILN in 2:1 configuration via open reduction and internal fixation (ORIF) with two double‐loop cerclage wires.

A 2:2 bolt configuration was used in 173/243 (71.2%) fractures. Alternative bolt distributions (Figure 4) included 2:1 in 12 (4.9%) cases, 1:2 in 11 (4.5%), 1:1 in 40 (16.5%), 2:0 in four (1.6%) and 1:0 in three (1.2%). Seven nails were placed in dynamic fashion with no bolts being used distally, three in ulnae, two in femora and one in a tibia. A total of 44 out of 852 (5.16%) possible cannulations were missed intraoperatively. A total of 39 (88.6%) of these were distal bolts (position 3 or 4). Out of these 44 missed cannulations, 36 (81.8%) were detected and retrieved intraoperatively while eight (18.2%) were only detected postoperatively. This leads to a total postoperative missed cannulation rate of eight out of 852, or 0.94%.

### 3.4 Follow‐up

Radiographic and clinical follow‐up was achieved for a total of 198 cases. The median duration of radiographic follow‐up was 57.5 days (range: 20–2693 days). For one case, clinical follow‐up was achieved 14 days postoperatively, but the case was lost to follow‐up thereafter. Unfortunately, 45 cases were lost to follow‐up from immediately postoperatively and were excluded from this part of the study.

Routine follow‐up involved rechecks at 3 and 6 weeks postoperatively, with additional follow‐up visits at three weekly intervals as necessary until clinical union was achieved. Out of 243 cases in this study, 136 followed this protocol. Mean time to clinical union for this population was 8.3 weeks (range: 1–24). An additional 16 cases were seen back a number of years postoperatively with bony union being documented at that time. Another 45 cases were seen back for at least one recheck and were considered to be progressing both clinically and radiographically as expected for that time point but were lost to further follow‐up prior to achieving clinical union. One case was lost to follow‐up following revision surgery – further detail is provided below.

When considering time to lameness resolution, this was specifically stated in 112 medical records. The mean time to lameness resolution for these 112 cases was 8.7 weeks (range: 3–44).

In four cases radiographic malalignment was noted immediately postoperatively necessitating revision under the same anesthetic episode. All four were femoral fractures. Three were initially stabilized via MINO while one severely comminuted fracture was stabilized via ORIF. Immediate revision resulted in acceptable alignment in all four cases.

Postoperative complications were encountered in 35/243 cases (14.4%); major in 13 cases (5.3%) and minor in 22 (9.1%). A total of 29 of these 35 complications (82.9%) were perioperative, occurring between 0 and 3 months postoperatively and 2/235 (5.7%) were short‐term occurring 3 to 6 months postoperatively. There were no mid‐term complications occurring 6–12 months postoperatively, but 4/35 (11.4%) complications were long‐term occurring after 12 months postoperatively. Major complications comprised biological slack (1), bolt fatigue (1), nail fatigue (1), seroma/distal bolt irritation (5) and surgical site infection (SSI) necessitating implant removal (5).

The case of biological slack was encountered in a German Shepherd Dog weighing 55.9 kg following tibial fracture stabilization. The proximal bolts both cut through the medial cortex leading to instability. An external skeletal fixator was placed 17 days postoperatively to address this. The case of bolt fatigue was in a Brittany Spaniel weighing 26.2 kg following use of a 5 mm nail placed in 2:2 configuration for stabilization of a comminuted humeral fracture. Both distal bolts failed resulting in collapse of the fracture and distal displacement of the nail into the supratrochlear foramen. The fracture healed despite the collapse, but the nail and retrievable bolt components were removed once clinical union had been achieved. The case of nail failure was in a 44 kg mixed breed dog where a 6 mm nail was used to stabilize a comminuted non‐reconstructable tibial fracture. Nail failure occurred 4 weeks postoperatively and was revised using a 7 mm nail, but the case was lost to follow‐up hereafter. Out of the five cases where distal bolts needed to be removed or shortened due to irritation, four were in the distal tibia while one was in the distal femur.

Minor complications included bolt fatigue with no revision required (7), SSI that responded to antibiotic therapy (10), delayed union (3) and seroma formation (2).

## 4 DISCUSSION

The results of this study indicate that the I‐Loc AS‐ILN system can be used successfully in a variety of fractures with a low complication rate. Satisfactory results were obtained across a wide spectrum of presentations comprising fractures affecting all long‐bones and of multiple configurations including epi−/metaphyseal and open fractures.

Over 60% of fractures in this case series were comminuted, with almost 30% of them being considered non‐reconstructable. In cases where the bony column cannot be, or is electively not reconstructed, the absence of load‐sharing leads to greater stresses being applied to the fixation system which must be accommodated through implant choice in order to avoid fatigue failure.^29^ Additionally, where small gaps remain, high strains can develop which lead to slower callus formation and delayed union.^30^, ^31^, ^32^, ^33^ Under such circumstances, the use of an intramedullary implant, placed near the neutral axis, is desirable as this will be protected from deleterious bending moments; in this regard interlocking nails or plate‐rod constructs are commonly used. This study indicates a low complication rate associated with such stabilization of non‐reconstructable fractures.

In this study, the major complication rate was 13/243 (5.3%) and the minor complication rate 22/243 (9.1%). Seroma formation secondary to distal bolt irritation was treated by bolt removal via stab incisions under sedation in radiography and as such, it is questionable whether this should be classified as a major complication. Excluding these from the major complication rate would reduce this to 3.3%. When comparing these complication rates to previously reported results following use of a plate‐rod construct,^34^ results following AS‐ILN use appear to be favorable. In the study by Reems et al.,^34^ a different classification method was used, with complications only being considered major if they had an effect on the final outcome for the dog. However, if they are reclassified based upon a requirement for revision surgery, as performed in this study, the major complication rate is 11/47 (23.4%) and the minor complication rate 8/47 (17%) which is substantially higher than that encountered with this AS‐ILN. Intramedullary pin migration accounted for 19% of these major complications. Due to the presence of locking bolts, in the absence of bolt failure, nail migration is not a complication that is encountered, and this may at least partially explain the lower complication rate noted in this study.

The major complication rate reported for this case series is also comparable to, or lower than, that reported for other AS‐ILN constructs. Again, differences in how complications were classified should be considered here and for the purposes of this discussion, to allow appropriate comparison, the complication rates from the paper detailing the results from the Surg'X nail have been reclassified as major or minor based upon the requirement for revision surgery so as to correlate with the results from the current study and the study regarding the Targon nail. Using this classification, the major complication rate reported with the Surg'X nail is 5/61 (8.2%),^18^ and with the Targon nail, is 9/57 (15.8%).^20^ The larger case numbers, which likely translates to increased experience with the I‐Loc system may be considered to contribute to this lower number; however, in the abstract previously presented where 100 cases stabilized using the I‐Loc nail were included, the major complication rate was similar, being only 3/100 (3%).^16^ As such, this low complication rate appears to be sustained, even within the earlier learning curve of using this system.

Out of 243 cases in this study, there were 15 SSIs, representing a 6.2% infection rate. Out of these, five went on to necessitate implant removal while 10 were managed using systemic antibiotics alone. Also, within this study, 23/243 (9.5%) fractures were classified as open. When these two case populations are examined, 14 out of the 15 SSIs documented (93.3%), developed in open fractures. Only one SSI developed in a dog with a closed fracture. Implant removal was achieved without difficulty in all five cases where this became necessary including three tibial fractures, one radius/ulna fracture and one femoral fracture. However, this does obviate the need to warn owners of the potential requirement for implant removal secondary to SSI, the risk of which appears to be substantially higher in dogs with open fractures. Overall, 21.7% of dogs with open fractures required AS‐ILN removal in this study, compared to 0.5% of dogs with closed fractures. This rate of implant removal correlates closely with that previously reported for open fractures stabilized using internal implants.^35^

Another major contributor to the complication rate reported here was irritation or seroma formation associated with the distal bolts, which was predominantly associated with tibial fractures; this accounted for five major and two minor complications. Similar complications have been reported with another AS‐ILN system previously.^20^ The paucity of soft tissue coverage in the distal tibia renders cutting the distal bolts short of critical importance. The majority of these specific complications occurred early within the case series, indicating that with awareness and meticulous technique, this complication should be avoidable. Interestingly, this complication has not been reported with the Surg'X nail. This may be because the cut tips of the I‐Loc bolts are sharper than the tips of the Surg'X bolts or could be because the Surg'X bolts are available in specific lengths rather than being cut to length intraoperatively.

The time to lameness resolution in this study was only available in 112 cases (46%) as in others this was not specifically stated in the records and could not be determined retrospectively. Subjectively, the time to lameness resolution of 8.7 weeks (range: 3–44 weeks) appears long and does not match clinical expectations. It is considered likely by the authors that the retrospective nature of the study contributed to this and skewed this data as the persistence of lameness may be more likely to be noted within the medical record than the absence of lameness. Additionally, owners may be more likely to return for longer‐term scheduled rechecks if a dog remains lame, as opposed to when lameness has resolved. However, these are simply speculations and prospective studies with standardized follow‐up times would be required to confirm or refute this suspicion.

The mean time to clinical union in this study was 8.3 weeks (range: 1–24). However, it should be noted that only patients that followed the recommended recheck schedule with rechecks at 3 and 6 weeks postoperatively, and additional follow‐up visits at three weekly intervals as necessary until clinical union was achieved were included in this calculation. While we feel that this is beneficial in that early and regular rechecks likely facilitated more accurate detection of when clinical union was achieved, it should be noted that this complicates comparison with other studies that follow a different schedule for rechecks.

Interlocking nails can be inserted and locked through incisions remote from the fracture site which preserves the extraosseous blood supply, fracture hematoma and periosteal blood supply.^8^ Simply by its nature as an intramedullary device, the interlocking nail facilitates fracture reduction, facilitates fixation by maintaining said reduction while bolts are placed, and facilitates maintenance of appropriate alignment in varus/valgus and pro/retrocurvatum.^8^ As such, AS‐ILNs lend themselves very well to minimally invasive osteosynthesis and MINO was employed in 91/243 (37%) of cases in this study. While alignment in two planes is controlled by the nail itself, rotational alignment does need to be carefully assessed. In four femoral fracture cases in this study, three of which were stabilized via MINO, unacceptable alignment with internal torsion of the distal femur, evidenced as excessive anteversion of the femoral head and neck relative to the distal femur, was noted immediately postoperatively necessitating an immediate return to surgery under the same anesthetic episode. Careful assessment of alignment intraoperatively is recommended, both when MINO is employed and when severely comminuted fractures are encountered in order to avoid this complication.

Within this retrospective canine series, 60/243 (24.7%) of cases were either metaphyseal or epiphyseal and as such would not have been considered appropriate indications for traditional interlocking nail use. However, the angular stability of the I‐Loc design,^15^ combined with the metaphyseal bolt location,^36^ render the I‐Loc system suitable for repair of such fractures, with positive results being reported in both feline,^17^ and now canine cases.

Additional design features that increase the adaptability of the I‐Loc system include the ability to alter bolt distribution, nail length, nail contour and bolt direction. While a 2:2 bolt configuration remained the most common in this series, alternative distributions were used in almost 30% of fractures. This flexibility facilitates avoidance of fracture lines or fissures that may extend to the regions of bolts 2 or 3. Additionally, in this facility and within this study, there is a trend toward using fewer bolts in fractures where rapid healing is anticipated in juveniles and in areas where load‐sharing has been achieved resulting in lower stresses being accommodated by the implant. In this study, nails were placed in a dynamic fashion in seven cases, with no bolts being used in the distal segment. This was always performed in reconstructable fractures that were stable to axial compression and in cases where prebending of the nail had been performed to follow natural pro‐ or retro‐curvatum, as this was felt to limit the impact of torsional forces as the nail would not be able to rotate within the medullary canal. The use of non‐locked or dynamic nails has been reported in specific situations within the human field without increasing complication rates^37^ but further research is needed in this area to determine precisely when bolt configuration should be altered.

Modification of the bullet‐nosed tip of the I‐Loc nail can be performed, as previously documented,^17^ which enables deep seating of the nail in very distal fractures. This was performed in 40/243 (16.5%) of cases in this study. While this was necessary in 17% of tibiae and 10% of femora, it was notable that nail shortening was deemed necessary in almost 50% (13 out of 27) of humeral fractures in order to achieve sufficient bolt purchase distally without the nail tip invading the supratrochlear foramen. This likely reflects the fracture patterns encountered at this facility as 10 of the humeral fractures concerned were classified as either distal metaphyseal or distal articular. It is not possible to state whether this distribution of fractures will be mirrored in other facilities, but it appears prudent to engage in careful preoperative planning, including ensuring provision for nail shortening when treating humeral fractures with the I‐Loc AS‐ILN.

Historically, the appropriateness of using interlocking nails in canine tibial fractures has been questioned. Concerns included that the introduction of the nail, cranial to the cranial cruciate ligament attachment, could result in an increase in tibial plateau angle, varus malalignment and iatrogenic cranial cruciate ligament damage. While, to date, no reports have substantiated these concerns, with one study showing no statistical difference in tibial plateau angle or valgus angle,^38^ nail prebending has been recommended to follow the natural tibial recurvatum in dogs and this was performed in 46/71 (65%) of tibial nails in this study. This percentage reflects a learning curve in clinical application. Chronologically, the first 23 tibial fractures treated in this series were all placed without being bent. Out of the next 48 tibial fractures, only two were placed without bending. It is the recommendation of the authors that all tibial nails should be bent to follow the natural recurvatum of the individual dog to avoid iatrogenic increases in tibial plateau angle. Prebending I‐Loc AS‐ILNs has also been shown to provide mechanical advantages by increasing their ability to resist bending resulting from eccentric compressive loads which may reduce the occurrence of tibial nail yield failure.^39^ Prebending has also been performed, albeit less frequently, in both the femur and the ulna in this case series. This is often a matter of surgeon preference, associated with a choice to anatomically reconstruct the fracture. By necessity, anatomical reconstruction will mandate prebending of the nail in both of these circumstances in order to accommodate the natural procurvatum.

When prebending is performed, most commonly for tibial fractures, this complicates use of the alignment guide for placement of the distal bolts. Following fracture reduction and nail insertion normograde from the stifle, the proximal bolts are placed routinely using the alignment guide. However, the bending of the nail will result in the alignment guide lying caudal to the distal metaphysis in these cases. In these cases, the distal bolts are inserted using a free hand technique. The alignment guide is used to determine the proximo‐distal location of the cannulations, while the cranio‐caudal location is identified along the bisector of the cranial and caudal tibial cortices.^8^ Intraoperative imaging is not commonly necessary.

Based on the results of this study, weight ranges appropriate for each nail diameter can be postulated as noted in Table 3. With a full spectrum of nails ranging from 3.0–8.0 mm, treatment of dogs ranging from 3.2 to 76.0 kg is possible. Care should be taken with the highest weight in the 8 mm nail category, as in that 76 kg dog, a LCP was placed in addition. If that dog is removed from analysis, the highest weight where the 8 mm nail was used in isolation was in a 68 kg St Bernard. Given the retrospective study design and the associated limitations, while these weight ranges can be used as a guideline, they should still be used with caution and combined with appropriate clinical decision‐making.

Due to the persistent rotational and bending instability associated with traditional interlocking nails,^3^, ^4^, ^6^, ^9^ supplementary implants were required to establish sufficient stability in up to 12% of cases.^10^, ^40^ While ancillary implants were used in 50/243 (21%) of cases in this series, none of these were employed to address persistent instability. The most commonly used ancillary implant was double‐loop cerclage wires which were utilized when anatomic reconstruction was elected. On the rare occasions when a lag or positional screw was used, they were employed to control a periarticular or articular fracture. Locking compression plates were used in two cases, one in a very large dog, where there was uncertainty whether the largest diameter interlocking nail alone would create a sufficiently robust construct, and one as dual bone stabilization in a radius/ulna fracture in conjunction with an AS‐ILN in the ulna. It is notable that two thirds of the supplementary implants used were in cases of metaphyseal or epiphyseal fractures, with just over half of epi−/metaphyseal fractures requiring ancillary fixation. This included all of the articular fractures. This is worthy of consideration when evaluating the positive results using the I‐Loc system for such fractures, as often this was a reflection of a combination of implants and not just the AS‐ILN itself. The only case that required supplementary fixation for persistent instability was the case that developed biological slack. The altered types and functions of the ancillary implants used in this case series reflect the angular stability achieved by the I‐Loc system, and the associated, aforementioned, increased applications.

With original interlocking nail designs, missed cannulations were reported ranging between 4% and 12%.^1^, ^22^ Modifications to the implantation technique for the I‐Loc AS‐ILN were made in order to limit the risk of off‐site distal bolt insertion. Namely, the systematic placement of temporary smooth locking posts from proximal to distal creates a rigid frame between the nail and alignment guide which reduces the alignment guide lever arm and its potential deviation from the nail axis. Should the cannulation be missed intraoperatively, as was the case in 5.16% of cannulations in this series, the standardized use of the feeler tool, the engagement of the smooth locking post, assessment for intraoperative slack, and the subsequent engagement of the bolt can all be used to detect errors intraoperatively. Combined, these tactics aim to substantially reduce the risk of missed cannulations, and indeed in this case series, the total postoperative missed cannulation rate was only 8/852 (0.94%), representing considerable improvement from original interlocking nail designs. Similarly low rates of missed cannulations have also been reported for the Surg'X system, where 5/365 (1.37%) of screws were missed intraoperatively, but most were reoriented intraoperatively resulting in a final missed cannulation rate of 1/365 (0.27%).^18^

This study had some limitations. The retrospective nature introduces potential sources of error, particularly with regard to the potential for reporting inaccuracies. Some of the effects of this will have been reduced by the reporting of a large number of cases and only including cases from a single institution but they still warrant consideration. The level of detail available within the records is also a limitation. At this facility, the use of clinical metrology instruments as a routine follow‐up method has not been embraced. A standardized lameness scoring method was not used amongst clinicians and goniometry was not consistently performed or documented. The ability to include such data for all included cases would certainly allow further and stronger conclusions to be drawn.

While follow‐up is standardized amongst surgeons at this facility, several cases were lost to follow‐up or did not attend follow‐up at scheduled times. If anything, the inconsistent follow‐up will likely have extended reported time to clinical union and time to lameness resolution and as such, it is considered unlikely that results have been over‐interpreted.

Additionally, it should be considered that this case series does include the initial learning curve of several surgeons with this technique. The authors consider this, to a degree, beneficial as it means that results should be applicable to other surgeons who have less experience with the instrumentation. It also facilitates comparison with the results of previous studies which detail early experience with the systems concerned.^18^, ^20^

In conclusion, positive clinical results were achieved using the I‐Loc AS‐ILN system in a wide variety of fractures including epi−/metaphyseal fractures and fractures of multiple configurations affecting any long‐bone provided sufficient space in the medullary canal exists to accommodate the nail. Accepting the limitations inherent to comparing these results to the historical literature, complication rates appear to be favorable to those associated with the plate‐rod system, non‐angle‐stable nails and other AS‐ILNs currently available. The weight ranges per nail diameter can be used as a guideline for implant selection followed by careful preoperative planning to determine when nail modifications may be beneficial based on the individual case.

## AUTHOR CONTRIBUTIONS

Perry KL, BVM&S, CertSAS, DipECVS, MSc, Vet Ed, FHEA, MRCVS: Designed the study, identified medical records, contributed cases, analyzed data, wrote and edited the manuscript. Wesslen R, LVT: Identified medical records, collected data and edited the manuscript.

## FUNDING INFORMATION

No grant or financial support was available for this study.

## CONFLICT OF INTEREST STATEMENT

The authors have no conflicts of interest to declare.

## Data Availability

The data that support the findings of this study are available from the corresponding author upon reasonable request.
